# Activating Mutations in *ERBB2* and Their Impact on Diagnostics and Treatment

**DOI:** 10.3389/fonc.2013.00086

**Published:** 2013-04-23

**Authors:** Grit S. Herter-Sprie, Heidi Greulich, Kwok-Kin Wong

**Affiliations:** ^1^Department of Medical Oncology, Dana-Farber Cancer InstituteBoston, MA, USA; ^2^Department of Medicine, Harvard Medical SchoolBoston, MA, USA; ^3^Department of Medicine, Brigham and Women’s HospitalBoston, MA, USA; ^4^Broad Institute of Harvard and MITCambridge, MA, USA; ^5^Ludwig Center at Dana-Farber/Harvard Cancer Center, Dana-Farber Cancer InstituteBoston, MA, USA; ^6^Lowe Center for Thoracic Oncology, Dana-Farber Cancer InstituteBoston, MA, USA

**Keywords:** ERBB2/HER2, activating somatic mutation, reversible and irreversible tyrosine kinase inhibitors, targeted therapies, resistance, lung cancer, breast cancer

## Abstract

Despite the ongoing “war on cancer,” cancer remains one of the major causes of human morbidity and mortality. A new paradigm of targeted therapies holds the most promise for the future, making identification of tumor-specific therapeutic targets of prime importance. ERBB2/HER2, best known for its role in breast cancer tumorigenesis, can be targeted by two types of pharmacological manipulation: antibody therapy against the extracellular receptor domain and small molecule compounds against the intracellular tyrosine kinase domain. Aberrant activation of ERBB2 by gene amplification has been shown to participate in the pathophysiology of breast, ovarian, gastric, colorectal, lung, brain, and head and neck tumors. However, the advent of next-generation sequencing technologies has enabled efficient identification of activating molecular alterations of *ERBB2*. In this review, we will focus on the functional role of these somatic mutations that cause ERBB2 receptor activation. We will additionally discuss the current preclinical and clinical therapeutic strategies for targeting mutationally activated ERBB2.

## Introduction

Rising incidences of neoplasia worldwide are estimated to translate into 13 million cancer deaths by 2030 (World Health Organization, 2012). In order to develop more effective and less toxic targeted cancer therapies, we must utilize our knowledge of malignant cell biology and design tailored antineoplastic compounds against diverse biological targets to supplement current standard treatment modalities, such as surgical resection, chemotherapy, and radiation therapy, to eradicate this frequently fatal disease. Although encouraging response rates are achieved in a few types of cancer with these standard treatment options, the majority of patients lack sufficient therapeutic options for long-term survival, especially those with advanced disease. Hence, additional therapies are urgently needed.

Because neoplastic cells frequently show “addiction” to mutationally activated oncogenes (Weinstein, 2002; Sharma and Settleman, 2010), such oncogenes comprise the most promising group of drug targets discovered to date. In the mid 1980s, the receptor tyrosine kinase (RTK) ERBB2 (also known as HER2 – Human Epidermal Growth Factor Receptor (EGFR) 2) was identified to be an oncogenic driver (Padhy et al., 1982; Bargmann et al., 1986; Di Fiore et al., 1987; Slamon et al., 1987). ERBB2 was first targeted with the monoclonal antibody, trastuzumab, which was approved by the Food and Drug Administration (FDA) in 1998. Although the addition of trastuzumab to chemotherapy significantly prolonged survival in patients with ERBB2-overexpressing breast or gastric cancers (Piccart-Gebhart et al., 2005; Romond et al., 2005; Joensuu et al., 2006; Bang et al., 2010), these clinical benefits failed to translate in improved survival of patients with ERBB2-overexpressing non-small cell lung cancers (NSCLCs) (Gatzemeier et al., 2004; Langer et al., 2004).

Oncogenic signaling by RTKs can also be abrogated by inhibition of tyrosine kinase activity with small molecules. Imatinib mesylate demonstrated proof of principle by successfully inhibiting constitutive signaling through the BCR-ABL fusion protein in chronic myelogenous leukemia (Druker et al., 2001). Additional tyrosine kinase inhibitors (TKIs) targeting various cellular signaling pathways have entered the clinic since imatinib mesylate was approved by the FDA in 2001, including inhibitors targeting ERBB2 in breast cancer (Geyer et al., 2006) and the related RTK, EGFR, in lung adenocarcinomas (Ku et al., 2011). The emergence of sophisticated genomic methodologies like next-generation sequencing enabled high-throughput detection of known and novel oncogenic mutations, and in particular revealed the presence of activating mutations of *ERBB2* in a variety of tumor types. These novel oncogenic alterations of *ERBB2* potentially offer unique therapeutic opportunities to a broader range of patients than previously anticipated by analysis of *ERBB2* amplification alone. However, it appears that it may be more difficult to successfully target *ERBB2* mutation than *ERBB2* amplification or *EGFR* mutation. Translation of this discovery to the clinic thus remains a major challenge.

## The ERBB/HER Receptor Family

The proto-oncogene *ERBB2* is a member of the ERBB/HER RTK family, additionally comprised of EGFR (EGFR/HER1/ERBB1), HER3/ERBB3, and HER4/ERBB4 (Hynes and Lane, 2005). Upon extracellular ligand binding, these four receptors mediate normal cell proliferation and cell survival via two major signaling pathways: Ras-Raf-MAPK and PI3K/Akt/mTOR. Whereas EGFR and ERBB4 have known extracellular ligands and possess active tyrosine kinase domains, no direct high-affinity ligand has been identified for ERBB2 (Carraway et al., 1994; Sliwkowski et al., 1994; Burgess et al., 2003). Furthermore, ERBB3 binds several different ligands, but has little or no tyrosine kinase activity, and is possibly able only to weakly autophosphorylate (Shi et al., 2010).

## Activation of ERBB2

Signaling specificity of each ERBB receptor is transmitted through unique patterns of C-terminal autophosphorylation sites (Olayioye et al., 2000; Yarden and Sliwkowski, 2001). Further complexity is added by receptor dimerization, which can occur either between two identical (homodimerization) or two different (heterodimerization) ERBB receptors. Under resting conditions, these cell surface receptors are found as monomers folded in a so-called “closed/tethered” autoinhibited conformation to prevent dimerization (Ferguson et al., 2003). Conformational rearrangement into an “open/extended” state occurs upon ligand binding to the extracellular domain. This process exposes the dimerization arm to establish the core of the dimer interface with a homologous region of a partner molecule. The extracellular dimeric structure facilitates reciprocal transactivation of the intracellular tyrosine kinase portions of each receptor. The uniqueness of ERBB2 among its family members is not only characterized by its inability to directly bind any known EGF family ligand, but also by being permanently fixed in the active conformation. Consequently, kinase autoinhibition to prevent uncontrolled receptor activation is not mediated by the ectodomain, but by a loop connecting the αC helix and β4 sheet within the kinase domain (Fan et al., 2008).

At least in part due to its constitutively active conformation, ERBB2 is the preferred dimerization partner for other ERBB family members. Although the existence of four receptors allows several different pairings and subsequently distinct patterns of downstream pathway engagement, ERBB2 heterodimers demonstrated increased potency in conveying extracellular signals (Yarden and Sliwkowski, 2001). It comes as no surprise that the most powerful signaling heterodimer – composed of ERBB2 and ERBB3 – functions as an oncogenic unit (Holbro et al., 2003; Hsieh and Moasser, 2007; Lee-Hoeflich et al., 2008). Lack of catalytic kinase activity does not prevent ERBB3 from heterodimerizing with other ERBB molecules. In fact, the primary oncogenic signaling apparatus of ERBB2-ERBB3 is crucial for activation of the PI3K/Akt pathway (Soltoff et al., 1994). Although ERBB2 possesses no direct docking sites for PI3K, ERBB3 mediates this process with six tyrosine binding sites for the regulatory subunit of PI3K (Prigent and Gullick, 1994; Soltoff et al., 1994). Indeed, clinical data by Tokunaga et al. (2006) shows positive correlation of ERBB2-expressing breast cancers and increased activation of Akt.

Three principal mechanisms of oncogenic activation of *ERBB2* have been identified to date: (i) amplification and overexpression, (ii) molecular alterations of the receptor, and (iii) inhibition of phosphatase activity (Ocana and Pandiella, 2013). Increased numbers of receptor molecules populating the cell surface increase the likelihood of dimerization and receptor tyrosine phosphorylation, even in the absence of ligand binding (Zhang et al., 2006; Endres et al., 2011). *ERBB2* overexpression or amplification was initially discovered in approximately one third of human breast cancers and is associated with more aggressive tumors and poorer outcome (Slamon et al., 1987). Other human tumor types have also been reported to harbor *ERBB2* amplification or overexpression, including lung cancers (Pellegrini et al., 2003; Langer et al., 2004), gastric cancers (Tanner et al., 2005; Bang et al., 2010), ovarian cancers (Tuefferd et al., 2007; Vermeij et al., 2008), prostate cancers (Minner et al., 2010), salivary gland tumors (Cornolti et al., 2007), and bladder cancers (Lae et al., 2010).

**Figure 1:**
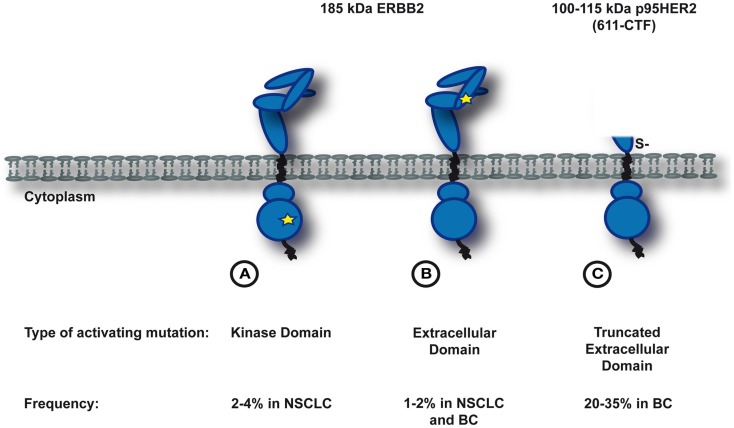
**Somatic activating mutations in *ERBB2***. Depicted is a simplified schematic of three known subclasses of ERBB2-mutants. **(A)** and **(B)** Activating mutations in the full-length protein. A star indicates the position of the activating mutation (**A** in the kinase domain, **B** in the extracellular domain). **(C)** Large deletions of the extracellular domain that yield the trucated form of ERBB2, p95HER2.

Mutational activation of ERBB2 can result from three types of somatic molecular alterations: small insertions and missense mutations in the kinase domain (Figure 1A), missense mutations in the extracellular domain (Figure 1B), or large deletions of the extracellular domain that yield the truncated form of ERBB2, p95HER2 (Figure 1C). The molecular characteristics, treatment opportunities, and potential mechanisms of resistance of these three classes will be discussed in the next sections.

Constitutive ERBB2 activation can also be achieved by insufficient dephosphorylation of the receptor. Although in rare cases ERBB receptors may transphosphorylate each other in the absence of ligand, overexpression, or mutations, intracellular phosphatases rapidly act as a fail-safe mechanism to dephosphorylate the receptor and terminate signaling (Ullrich and Schlessinger, 1990). Recently, studies by two different groups provided the first evidence that phosphatase activity is essential to control oncogenic ERBB2 signaling. Sun et al. (2011) demonstrated that mutational inactivation of the phosphatase PTPN12 caused activation of ERBB2 in triple negative breast cancer cell lines. Similarly, Vermeer et al. (2012) analyzed breast cancer cell lines to understand the correlation between decreased PTPN13 expression and poorer overall survival. The authors found a novel signaling complex consisting of ERBB2 and EphrinB1 which is regulated by transient association with PTPN13 and Src. In absence of the phosphatase PTPN13, activated Src associates with the ERBB2 kinase domain and phosphorylates EphrinB1, which induces Erk1/2 phosphorylation (Vermeer et al., 2012).

## ERBB2 Mutations in Carcinogenesis

The clinical success of gefitinib, an inhibitor of EGFR, in a subset of lung cancers harboring activating mutations within the kinase domain of EGFR led to the investigation of analogous mutations of *ERBB2*. ERBB2 kinase domain mutations were found to occur in 2–4% of lung adenocarcinomas (Stephens et al., 2004; Shigematsu et al., 2005; Buttitta et al., 2006) and cause increased survival, invasiveness, and tumorigenicity in cell-based transformation assays (Wang et al., 2006). Similarly to NSCLC driven by *EGFR* mutation, the clinical and pathological characteristics of patients with *ERBB2* mutations have been attributed to patients of the female sex, Asian ethnicity, never-smoker status, and adenocarcinoma subtype. However, a recent study of 1,478 U.S. patients with lung adenocarcinomas found no association of *ERBB2* mutation with sex and race, but confirmed that mutations of the kinase domain of *ERBB2* are mutually exclusive with *EGFR*, *KRAS*, and *ALK* mutations (Arcila et al., 2012).

The most prevalent alteration of *ERBB2* involves the in-frame insertion/duplication A775_G776insYVMA within exon 20, a sequence also present in exon 20 of the *EGFR* gene (Stephens et al., 2004). Similar in-frame insertion mutations were also identified in ovarian cancers (Anglesio et al., 2008). These insertions induce a conformational change of the autoinhibitory αC-β4 loop, thus narrowing the ATP-binding cleft and promoting enhanced kinase activity (Gazdar et al., 2004; Fan et al., 2008). *In vitro* studies have shown that this ERBB2-mutant potently transphosphorylates EGFR in the absence of ERBB ligands rendering EGFR susceptible for dimerization (Wang et al., 2006). Single nucleotide missense substitutions of this region of *ERBB2* have also been reported in breast cancer, gastric cancer, and colorectal cancer (Lee et al., 2006b).

Although oncogenic tyrosine kinase mutations frequently alter the ATP-binding pocket, we recently identified an alternate mechanism of ERBB2 activation resulting from extracellular domain mutations that cause reduction-sensitive covalent dimerization (Greulich et al., 2012). These missense substitutions cluster in subdomain II, a region characterized by 11 disulfide bonds (Cho et al., 2003), and impact intramolecular disulfide bond formation (Greulich et al., 2012). Mutation of cysteine residues in this region that participate in intramolecular disulfide bonds, such as S335C (Greulich, unpublished observation), or mutation of residues important to stabilization of disulfide-bonded loops, such as G309E, can both promote intermolecular disulfide bond formation, resulting in constitutively dimerized and activated ERBB2 (Greulich et al., 2012).

Reduction-sensitive dimerization is not the only mechanism by which ERBB2 extracellular domain mutations constitutively activate enzymatic activity; ERBB2 S310F and S310Y mutations, found in 1–2% of lung cancers and breast cancers, behave more similarly to the ERBB2 kinase domain mutants in that they cause elevated C-terminal tail phosphorylation without evidence of covalent dimerization. Of note, the S310F lesion was also detected in 1/316 ovarian cancers (Cancer Genome Atlas Research Network, 2011) and in a bladder cancer cell line, 5637 (Barretina et al., 2012).

Whereas activating mutations within the kinase domain of ERBB2 show close homology to their counterparts within EGFR, the extracellular domain mutations are not as closely mirrored. Oncogenic mutations affecting the ectodomain of *EGFR* have been identified in subdomain I, II, and IV (Lee et al., 2006a). Although the mechanism of receptor activation has not yet been characterized for these EGFR extracellular domain mutations, it is tempting to speculate that the underlying tumorigenic mechanism is caused by a less tethered conformation of the extracellular domain as most amino acid substitutions localize to interdomain interfaces (Lee et al., 2006a).

The third type of mutant ERBB2 is structurally different from the first two, as these derivatives lack substantial parts of the extracellular domain and are termed p95HER2 or HER2 carboxyl terminal fragments (CTF) (reviewed in Arribas et al., 2011). These truncated ERBB2 proteins have been predominantly found in breast cancers and cause resistance to trastuzumab (Molina et al., 2002; Scaltriti et al., 2007). Only a few cases of lung adenocarcinoma were reported to harbor these mutations (Cappuzzo et al., 2012). Two distinct mechanisms yield p95HER2 fragments: alternative mRNA translation from internal initiation codons (positions 611 and 678, respectively) and proteolytic shedding of the ectodomain of the full-length receptor (Christianson et al., 1998; Anido et al., 2006). Strikingly, *in vitro* studies of the membrane-anchored p95HER2 fragment known as 611-CTF (100–115 kDa) revealed more rapid and acute activation of different signaling pathways compared with the full-length receptor and the 648-CTF fragment (Pedersen et al., 2009). Additionally, this hyperactive p95HER2 fragment was shown to promote more aggressive and metastatic breast cancer progression by induction of a specific gene set (Pedersen et al., 2009). The pathological features attributed to overexpression of 611-CTF are postulated to be a result of its short extracellular domain, which contains five cysteines. Thus again, constitutive generation of activated homodimers is assumed to be maintained by intermolecular disulfide bonds (Pedersen et al., 2009).

## ERBB2 as a Therapeutic Target

Two different strategies for targeting ERBB2 have successfully entered the clinic: antibodies directed against the extracellular domain of the receptor, and small molecule TKIs acting on the intracellular kinase domain.

The mechanism of action of monoclonal antibodies toward ERBB2-overexpressing cancer cells include removal of ERBB2 from the cell surface by endocytosis to diminish intracellular signaling, and induction of an immune system-mediated antitumor response. Several ERBB2-directed monoclonal antibodies have been developed, including trastuzumab and pertuzumab. Whereas trastuzumab interacts with subdomain IV of the extracellular domain (Cho et al., 2003), pertuzumab binds subdomain II, which harbors the dimerization arm and thus inhibits receptor dimerization (Franklin et al., 2004). Trastuzumab can also be conjugated to DM1, an inhibitor of tubulin polymerization derived from maytansine, to efficiently deliver DM1 to ERBB2-overexpressing cancer cells (Lewis Phillips et al., 2008). Despite promising preclinical data, clinical development of ertumaxomab, a bispecific antibody capable to bind mature T cells and ERBB2, was discontinued (Kiewe et al., 2006).

Small molecule TKIs are typically competitive inhibitors, preventing ATP from binding to its natural site within the kinase region due to the higher affinity of the TKI for the ATP-binding pocket. ERBB family TKIs fall into two categories: reversible inhibitors, like erlotinib, gefitinib, and lapatinib, that can be released from the receptor; and irreversible inhibitors, such as afatinib, neratinib, pelitinib, and dacomitinib, that covalently modify the receptor. Although the *in vitro* efficacy of the irreversible inhibitors was demonstrated to be superior to that of reversible inhibitors, irreversible ERBB blockade requires biosynthesis for receptor recovery, both a benefit and a drawback (Sanchez-Martin and Pandiella, 2012).

From the oncologist’s point of view, irreversible inhibition is highly desired for tumor control. However, ERBB signaling is also vital to non-malignant tissues, and inhibition of ERBB2 is associated with unwanted toxicities. For instance, trastuzumab can provoke cardiotoxicity, especially when administered in combination with anthracyclines (Slamon et al., 2001). Thus, careful evaluation is required prior to utilization of more potent irreversible inhibitors, which may result in increased toxicity. It is possible that non-competitive inhibitors could serve as a valuable alternative, particularly to combat eventual resistance to current TKIs (Ocana and Pandiella, 2013).

Despite robust preclinical and encouraging clinical data in various cancer types, a third class of antineoplastic agents active against ERBB2, HSP90 inhibitors, has still not been approved by the FDA. HSP90 is a chaperone that governs the conformational maturation and folding of ERBB2. Inhibition of HSP90 leads to ubiquitylation and proteasomal degradation of ERBB2 and its downstream signaling partners. In a Phase II study, combination treatment with trastuzumab and the HSP90 inhibitor tanespimycin (also known as 17-AAG) was demonstrated to be active in patients with breast cancer who had progressed on trastuzumab therapy (Modi et al., 2011).

Given our current knowledge of the biology of activating mutations of ERBB2, single agent antibody-based treatment strategies may be of limited clinical relevance. In particular, truncated p95HER2 fragments naturally evade antibody binding due to the absence of the extracellular domain and binding of trastuzumab to ectodomain- or kinase domain-mutated ERBB2 forms presumably fails to prevent ligand-mediated ERBB3-ERBB2 signaling (Agus et al., 2002). Our *in vitro* data furthermore indicates that, whereas survival of Ba/F3 cells expressing mutants of G309 and S310 was effectively inhibited upon trastuzumab treatment, other ectodomain-mutants were less responsive (Greulich et al., 2012). Further *in vivo* investigation will be required to determine response in a more physiological setting. Additionally, it would be of interest to evaluate whether pertuzumab is able to bind and impact survival of cancer cells expressing ectodomain-mutants. It remains unclear whether combinatorial treatment of trastuzumab and pertuzumab would be effective, given recent data obtained from ERBB2-positive metastatic breast cancer (Baselga et al., 2012).

Importantly, tissue-specific properties may hamper therapeutic success of antibody-based treatment schedules. Whereas trastuzumab has recently been approved for the treatment of metastatic gastric cancer in combination with cytotoxic agents (Bang et al., 2010), similar studies targeting overexpressed/amplified *ERBB2* in NSCLC (Gatzemeier et al., 2004; Langer et al., 2004; Lara et al., 2004b; Zinner et al., 2004; Krug et al., 2005; Herbst et al., 2007) and prostate cancer (Morris et al., 2002; Lara et al., 2004a; Ziada et al., 2004) have reported modest or disappointing results. It remains to be determined whether this primary resistance to trastuzumab results from inaccessibility of the receptor. For example, Nagy et al. (2005) found that MUC4, a membrane-associated mucin, masked the extracellular domain of ERBB2. In light of this finding, evaluation of MUC4 overexpression as a possible mechanism for primary resistance to trastuzumab in NSCLC should be done. Indeed, 80–85% of NSCLCs express MUC4, and adeno- and adenosquamous-carcinomas are characterized by high levels of MUC4 expression (68 and 75%, respectively) (Kwon et al., 2007). Further analyses by Karg et al. (2006) suggest that *MUC4* and *ERBB2* expression are positively correlated and might be involved in the repression of apoptosis and differentiation. However, primary resistance to trastuzumab in prostate cancer may involve other mechanisms, as *MUC4* expression was not detectable in malignant prostate tissue (Cozzi et al., 2005).

The identification of activating mutations within the kinase domain of ERBB2 offered an additional therapeutic possibility (Stephens et al., 2004). EGFR-TKIs were shown to be ineffective against ERBB2 mutations (Wang et al., 2006; Cappuzzo et al., 2007; Engelman et al., 2007). By contrast, several ERBB2-directed TKIs showed effective anti-proliferative properties. Despite promising preclinical data with neratinib (HKI-272; an irreversible ERBB inhibitor of EGFR and ERBB2) in the ERBB2-mutant NCI-H1781 cell line (Shimamura et al., 2006), clinical evaluation in patients with EGFR-driven lung adenocarcinomas does not support use of this inhibitor as a single agent (Wong et al., 2009; Sequist et al., 2010). However, three of four patients harboring the rare *EGFR* mutation G719X (X indicates substitution of glycine by either serine, cysteine, or alanine) were found to respond to neratinib (Sequist et al., 2010). Strikingly, neratinib showed promising activity in ERBB2-overexpressing breast cancers and could potentially be approved as a first-line therapy in locally advanced or metastatic ERBB2-overexpressing breast cancers (Chow et al., 2009; Limentani et al., 2009; Burstein et al., 2010; Awada et al., 2013).

Preclinical activity for afatinib (BIBW2992), a second irreversible inhibitor of EGFR and ERBB2, was demonstrated in Ba/F3 cells expressing an ERBB2-mutant with an insertional mutation at codon 776 and in transgenic lung cancer models (Li et al., 2008). De Greve et al. (2012) recently provided the first evidence of clinical benefit from treatment with afatinib. In this study, patients were initially diagnosed with lung adenocarcinoma harboring exon 20 *ERBB2* mutations and had progressed under various antineoplastic regimes. Three of five such identified patients were eligible for treatment response evaluation and two patients showed rapid metabolic response within 1–2 weeks. Although single agent afatinib did not extend overall survival in patients with advanced, metastatic NSCLC after failure of other therapeutic options (Miller et al., 2012), it did prolong progression-free survival and it appears rational to investigate the synergistic effects of afatinib and paclitaxel in this patient population. Furthermore, our preclinical data utilizing an inducible mouse model of mutant *ERBB2* (A775_G776insYVMA) in lung epithelium revealed that the combination of afatinib and an mTOR inhibitor (rapamycin) were effective in mediating tumor shrinkage (Perera et al., 2009). Thus, combinatorial treatment approaches are likely to positively influence clinical outcome.

Dacomitinib (PF00299804), a third irreversible pan-ERBB inhibitor, is currently under clinical investigation due to promising preclinical studies (Engelman et al., 2007; Janne et al., 2011). The rationale for irreversible TKI development to fight ERBB2-activating mutations originates from experience with reversible TKIs targeting EGFR-activating lesions. Despite their initial response, almost all of these cancers rapidly develop resistance and result in little overall survival benefit (Maemondo et al., 2010). In about half of these resistant cancers, a secondary mutation within the catalytic cleft of the kinase domain is responsible for ineffective reversible drug activity and subsequent oncogenic proliferation (details in Section Mechanisms of Resistance).

Following discovery of activating mutations within the ERBB2 extracellular domain, we analyzed the growth inhibitory effects of neratinib, afatinib, and lapatinib in Ba/F3 cells expressing the variant mutants (Greulich et al., 2012). Effective abrogation of cell survival was observed for all three inhibitors; however, the reversible inhibitor lapatinib was 5- to 10-fold less effective than neratinib and afatinib. Cells expressing the ectodomain-mutants were consistently more sensitive to these inhibitors than cells expressing the kinase domain mutant, A775_G776insYVMA.

Thus far, we focused our review on preclinical and clinical studies evaluating the existing anti-ERBB2 agents on cancers harboring activating mutations of ERBB2 as single agents with or without adjacent chemotherapy. However, the ERBB2 signaling cascade plays a pivotal role in oncogenesis and obviously affects a multitude of other key signaling nodes. Thus, combination of different ERBB2-directed agents (antibody + TKI) or with other targeted therapies (HSP90 inhibitors, MEK inhibitors, mTOR inhibitors, etc.) present valid options to combat ERBB2-driven oncogenesis.

Recent clinical data showed a significant overall survival benefit of patients with heavily pretreated metastatic ERBB2-positive breast cancer upon dual ERBB2 blockade through trastuzumab and lapatinib (Blackwell et al., 2012). Further studies are warranted to confirm the superiority of this cytotoxic agent-free regiment in earlier clinical settings. Another interesting treatment approach of synergistic efficacy was presented by Garcia-Garcia et al. (2012). The authors analyzed five different cell lines resistant to trastuzumab and lapatinib. The combination treatment of lapatinib and INK-128, an mTOR inhibitor, induced increased apoptosis in both *in vitro* and *in vivo* experiments (Garcia-Garcia et al., 2012). Along the same line, a Phase I study of neratinib and temsirolimus, an mTOR inhibitor, demonstrated encouraging antitumor activity in patients with ERBB2-overexpressing NSCLCs and breast cancers (Gandhi et al., 2011).

Although activating mutations of ERBB2 were identified in various tumor types and several potential therapeutic options are at hand, specific screening for these lesions has not been translated into clinical routine yet.

## Mechanisms of Resistance

Despite the plethora of ERBB2 targeted compounds, we currently lack a sound understanding why tumor shrinkage is short-lived and only a relatively small percentage of patients benefit from these therapies. Major mechanisms of primary and acquired resistance to anti-ERBB therapeutics include (reviewed in Tortora, 2011): (1) alteration of the extracellular domain, including missense substitutions to impede epitope recognition, masking of epitopes, or expression of ectodomain-truncated ERBB2 fragments; (2) second-site mutations in the RTK domain; (3) overexpression of alternative ERBB ligands or receptors to counteract for receptor inhibition; (4) alternative signaling from other receptors such as the insulin-like growth factor-1 receptor (IGF1R) or MET; (5) aberrant signaling caused by downregulation (p27) or loss (PTEN) of downstream controllers; and (6) aberrant activation of secondary downstream growth and survival pathways, such as Ras-Raf-MAPK, PI3K/Akt/mTOR.

**Figure 2:**
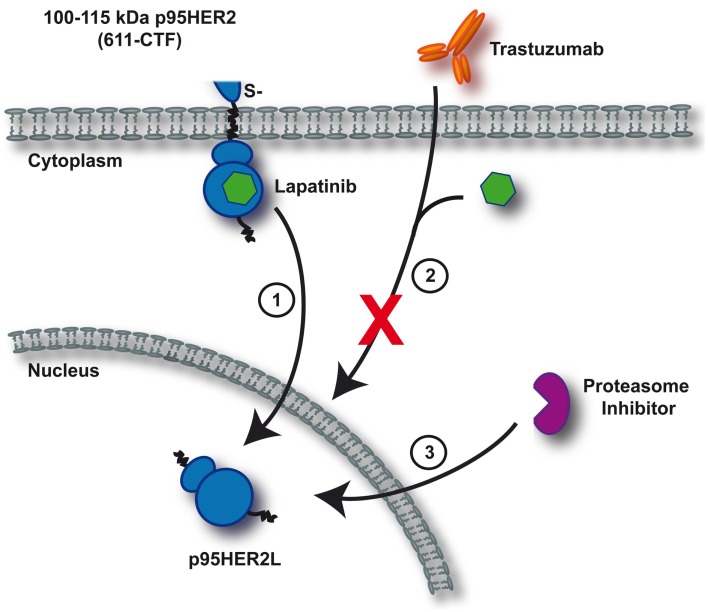
**Mechanism of resistance upon continuous lapatinib treatment**. Depicted is a simplified schematic of lapatinib-induced resistance toward current anti-ERBB2 therapeutics as identified by Xia et al. (2011). Continuous inhibition of 611-CTF with lapatinib induces nuclear p95HER2L expression (1). Trastuzumab and lapatinib are ineffective in targeting nuclear p95HER2L, thereby failing to control oncogenic proliferation (2). As formation of p95HER2L potentially depends on proteasomal processing, proteasome inhibition effectively prevents p95HER2L emergence (3).

Retrospective studies on tumors expressing truncated p95HER2 fragments revealed that these tend to be resistant to any current therapeutic antibody approach as the required epitopes are missing (Scaltriti et al., 2007; Sperinde et al., 2010). However, two independent groups recently generated monoclonal antibodies that specifically recognize 611-CTF (Parra-Palau et al., 2010; Sperinde et al., 2010). Conceivably, this novel diagnostic tool is of valuable clinical relevance because it allows discrimination of which patients will benefit from antibody-based therapies and which will be resistant. As the studies performed by Scaltriti et al. (2010) convincingly demonstrated that treatment with lapatinib effectively inhibits p95HER2, improved treatment stratification is available for patients harboring this particular activating ERBB2 mutation. Regardless, caution is still warranted since experiments by Xia et al. (2011) revealed that chronic lapatinib treatment is capable of inducing nuclear expression of truncated ERBB2, thereby escaping further therapeutic effectiveness. Additionally, formation of nuclear lapatinib-induced p95HER2 was blocked upon proteasome inhibition (Figure 2) (Xia et al., 2011). It remains to be tested whether this phenomenon is also relevant if: (1) full-length ERBB2 is targeted, or (2) any of the irreversible TKIs employs a similar strategy to evade antitumor control.

Analogous to a commonly observed event during TKI treatment of EGFR-driven lung adenocarcinomas, lapatinib application was shown to induce secondary mutations within the ERRB2 kinase domain consequently leading to TKI resistance. *In vitro* analyses identified three point mutations, L755S, L755P, and T798M to confer resistance to lapatinib (Kancha et al., 2011). Threonine 798 is the ERBB2 “gatekeeper” residue that is located at the periphery of the nucleotide-binding site of ERBB2 kinase (Aertgeerts et al., 2011), and regulates access to a deep hydrophobic pocket in the active site (Schindler et al., 2000). This event is analogous to replacement of threonine 790 with methionine (T790M) in erlotinib-resistant lung adenocarcinoma. The gatekeeper mutation enhances the affinity of the oncogenic form of the receptor for ATP, allowing continued proliferation in the presence of the drug (Yun et al., 2008). The potential of irreversible EGFR/ERBB2 inhibitors to overcome drug resistance due to gatekeeper mutations was recently demonstrated *in vitro* and *in vivo* (Kobayashi et al., 2005; Engelman et al., 2007; Minami et al., 2007; Li et al., 2008; Zhou et al., 2009).

## Concluding Remarks

During the past decades, the ERBB2 signaling cascade gained significant importance in the oncogenesis of many tumor types. The discovery of primary activating mutations and the emergence of acquired secondary mutations represent sophisticated challenges for effective treatment approaches. Our next steps in evaluating potential ERBB2-directed therapeutics clearly rely on: adequate diagnostic properties for specific patient selection and identification of tissue-specific mechanisms of resistance to initiate well-designed clinical trials of combinational treatment strategies.

## Conflict of Interest Statement

The authors declare that the research was conducted in the absence of any commercial or financial relationships that could be construed as a potential conflict of interest.
